# Clinical Evaluation of Amniotic Membrane as an Adjunct to Modified Coronally Advanced Flap and Resin‐Modified Glass Ionomer Restoration in the Management of Gingival Recession Associated With Noncarious Cervical Lesions: A Randomized Split Mouth Trial

**DOI:** 10.1155/ijod/1839674

**Published:** 2026-10-19

**Authors:** S. Sanidhya, Deepa G. Kamath, Mranali K. Shetty, Aradhya Sinha, N. Srikant

**Affiliations:** ^1^ Department of Periodontology Manipal College of Dental Sciences Mangalore Manipal Academy of Higher Education Manipal India manipal.edu; ^2^ Department of Oral Pathology Manipal College of Dental Sciences Mangalore Manipal Academy of Higher Education Manipal India manipal.edu

**Keywords:** amnion membrane, gingival recession, modified coronally advanced flap, noncarious cervical lesion, perioplastic surgery, regenerative surgery, resin-modified glass ionomer cement

## Abstract

**Introduction:**

Gingival recession often coexists with noncarious cervical lesions (NCCLs), complicating treatment outcomes. This study assesses the efficacy of modified coronally advanced flap (MCAF) and resin‐modified glass ionomer cement (RMGIC), with or without amniotic membrane (AM), in enhancing root coverage and clinical results in gingival recession associated with NCCLs through a randomized trial.

**Materials and Methods:**

A total of 15 systemically healthy patients (≥18 years), irrespective of gender, presenting with Pini‐Prato et al. Class B− or B+ surface defects associated with Miller’s Class I or II gingival recession, were selected. Eligible sites included maxillary posterior teeth per quadrant. A split‐mouth randomized clinical trial over 6 months compared MCAF + AM + RMGIC versus MCAF + RMGIC in NCCL‐associated recession. Mean standard deviation for the clinical variables was calculated for each parameter. Paired *t*‐test was used for intragroup comparison and independent *t*‐test for intergroup comparisons.

**Results:**

The test group consistently demonstrated superior clinical outcomes across all parameters compared to the control. Significant improvements were observed in probing depth (PD), clinical attachment level, recession width (RW), keratinized tissue height (KTH), gingival thickness (GT), and esthetic score. The use of AM adjunctively enhanced healing, root coverage, and overall treatment efficacy at 6 months.

**Conclusion:**

The combination of MCAF, RMGIC, and AM significantly enhances root coverage, esthetic outcomes, and periodontal stability. This less invasive approach offers a promising alternative to traditional techniques, warranting further investigation for long‐term clinical application and protocol optimization.

**Trial Registration:** ClinicalTrials.gov identifier: REF/2023/ 02/064206

## 1. Introduction

Gingival recession is a widespread dental disorder marked via apical displacement of gingival edge, resulting in the revealing of tooth roots. This may lead to several consequences, such as dentin hypersensitivity, cosmetic issues, and also heightened chance of root cavities. Gingival recession is prevalent, with research showing that it affects between 50% and 85% of adults at some stage in their lives [1, 2]. Factors adding to gingival recession encompass spontaneous damage due to vigorous tooth brushing, periodontal infection, anatomical predilection, and lifestyle choices.

Noncarious cervical lesions (NCCLs) are characterized by the loss of dental hard tissue at the cementoenamel junction (CEJ) in the absence of carious activity. NCCLs may result from several etiological features, like erosion, abfraction, and abrasion. These lesions frequently arise alongside gingival recession, resulting in a complicated clinical situation that complicates successful therapy. The existence of NCCLs frequently complicates the therapy of gingival recession, as they might obstruct optimum root coverage and influence the stability of surgical results [3, 4].

The correlation between NCCLs and gingival recession is significant; almost 50% of gingival recessions are linked to NCCLs [5]. This connection requires a multidisciplinary treatment approach that integrates restorative and periodontal treatments to attain optimal outcomes. Diverse therapeutic approaches have been suggested for addressing gingival recession linked to NCCLs. Surgical methods like the coronally advanced flap (CAF) are deemed helpful pro attaining root coverage. The CAF approach entails coronal relocation of gingival tissue in uncovered root side, hence improving esthetics and diminishing discomfort. Nevertheless, the presence of NCCLs may necessitate further restorative interventions to rectify the loss of tooth structure. Resin‐modified glass ionomer cement (RMGIC) has become an effective restorative material for addressing NCCLs owing to its distinctive characteristics. RMGICs exhibit superior adherence to dental tissue while releasing fluoride, contributing to caries prevention [6]. Studies have shown that RMGICs can successfully restore NCCLs while permitting following surgical tehnique, like CAF, to attain root coverage [1, 3]. In a report of Santamaria et al. [4], modified CAF (MCAF) procedure signifies an advancement of conventional CAF procedures. MCAF integrates alterations designed to enhance surgical results, especially in instances involving NCCLs. This strategy enhances flap adaptation and improves predictability in attaining root coverage [4].

Recent breakthroughs in regenerative dentistry have incorporated amniotic membrane (AM) as an auxiliary material in periodontal procedures. The AM exhibits distinctive biological characteristics that facilitate healing and tissue regeneration. Its anti‐inflammatory and immunomodulatory properties can improve surgical results by promoting expedited healing and minimizing postoperative problems. The incorporation of AM with surgical methods like MCAF may offer enhanced advantages in addressing gingival recession linked to NCCLs. The application of AM may enhance clinical outcomes by facilitating superior tissue integration and improving root coverage. Due to the complexity of managing gingival recession linked to NCCLs, substantial study is required to assess different treatment approaches. The proposed investigation is to measure the effectiveness of the amnion membrane plus MCAF and RMGIC restoration against MCAF and RMGIC restoration. Thus, the present study helps to elucidate optimum treatment techniques for dentists addressing these complex illnesses by examining various approaches through a randomized clinical trial methodology [3, 5, 6].

Based on this discussion, the study aims to evaluate and compare clinical effectiveness and esthetic outcomes of MCAF with RMGIC with or without AM in treating gingival recession associated with NCCLs.

## 2. Materials and Methods

### 2.1. Study Setting

1. Participants for this study were chosen from the Outpatient Department (OPD) of Periodontics at Manipal College of Dental Sciences, Mangalore. The study included individuals of all genders. The study received ethical approval from the Human Subjects Ethics Board (Reference Number 23019) (Clinical trials registry, dated February 28, 2023), of MCODS, Mangalore, Manipal Academy of Higher Education, Manipal, in accordance with the 2000 revision of the 1975 Helsinki Declaration.

### 2.2. Study Design

The study was a prospective, randomized split‐mouth design clinical study performed over 6 months period. The treatment allocation was determined by a simple coin‐toss method immediately before surgery. The left or right surgical site was randomly allocated to the respective treatment group, with the allocation generated immediately before the procedure. The randomization outcome was documented in the study records, and the surgeon was informed of the allocation only immediately before surgery.

Test group: MCAF technique using amnion membrane and resin‐modified GIC in Pini‐prato et al. [6] class B− and class B+ of surface defects in teeth associated with Miller’s Class 1 or Class 2 recession of gingiva.

Control group: MCAF method utilizing resin‐modified GIC in Pini‐prato et al. [6] class B− and class B+ of surface defects in teeth associated with Miller’s Class 1 and Class 2 recession of gingiva.

### 2.3. Sample Size

The sample size calculation was based on the primary clinical outcome reported in Table 1 of the reference article. A clinically significant difference of 2 units was considered, based on the values reported in the reference. Using a 5% alpha error and 90% study power, with the corresponding variability reported in Table 1, the required sample size was calculated as 15 subjects per group. Thus, the sample size was considered adequate to detect a clinically meaningful difference in the primary outcome.

**Table 1:** Comparison of mean plaque index scores between the two groups at baseline and after 6 months.

Group	Time point	*N*	Mean ± SD	Mean difference ± SD	*t*	*p*‐Value
Intragroup comparison:						
Test	At baseline	15	2.56 ± 0.15	1.43 ± 0.11	50.24	<0.001 ^∗^
	After 6 months	15	1.13 ± 0.2			
Control	At baseline	15	2.63 ± 0.42	1.07 ± 0.15	27.01	<0.001 ^∗^
	After 6 months	15	1.56 ± 0.39			
Intergroup comparison:						
Test	At baseline	15	2.56 ± 0.15	0.07 ± 0.5	0.53	0.605
Control	At baseline	15	2.63 ± 0.42			
Test	After 6 months	15	1.13 ± 0.2	0.43 ± 0.46	3.61	0.003 ^∗^
Control	After 6 months	15	1.56 ± 0.39			
Test	Mean difference	15	1.43 ± 0.11	−0.36 ± 0.2	−6.81	<0.001 ^∗^
Control	Mean difference	15	1.07 ± 0.15			

*Note*:  ^∗^statistically significant difference (*p*  < 0.05).

N=2  Z12 −α/+Z1−β2σ2d2.

*N* represents number of samples, *d* represents the minimum difference in values which make clinically related effect, and *s* is the average standard deviation. *Z*(1–a/2): *Z*‐score for alpha error chosen, *Z*(1–*b*): *Z*‐score for power chosen.

### 2.4. Inclusion Criteria

1. The participants who signed informed consent were eager to take part in this study.
2. Participants above 18 years.
3. Individuals meeting the criteria outlined by Pini‐Prato et al. [6] for class B− and class B + surface defects in teeth, along with gingival recession, affecting teeth on either side of the midline ranging from central incisors to molars in the maxilla.
4. Miller’s Class I and Class II gingival recession is included.
5. Plaque score of full dentition should be less than 1.
6. Mombelli’s sulcular bleeding score of full mouth should be less than 20%.
7. Cervical abrasion was devoid of restoration.

### 2.5. Exclusion Criteria

1. Compromised systemic illness
2. Pregnancy
3. History of root coverage
4. Cervical abrasion with restoration
5. Patient in need of antibiotic prophylaxis
6. Smoking
7. Buccal and lingual inclination of teeth
8. Undergoing fixed orthodontic therapy
9. Mobile or fully restored teeth.

## 3. Clinical Measurements

### 3.1. Indices

1. Plaque Index (PI): Plaque accumulation was assessed using the Silness and Löe Plaque Index, which evaluates plaque thickness on the facial, lingual, mesial, and distal surfaces of each tooth using a 0–3 scoring system, with higher scores indicating greater plaque accumulation.
2. Modified sulcular bleeding index (SBI) by Mombell et al. [7].

A modified SBI is assessed on labial/buccal and lingual surfaces of all teeth by scoring criteria.

### 3.2. Clinical Measurements

The following clinical parameters are assessed on the buccal/labial aspect of all study teeth at baseline and 6 months after surgery [8].

1. Relative gingival recession (RGR): Measured as the distance from gingival margin (GM) to apical border of the stent.
2. Recession width (RW): RW is assessed tangentially at the midpoint of the facial CEJ.
3. Probing depth (PD): Calculated from GM to gingival sulcus.
4. Relative clinical attachment level (RCAL): Measured as the total of RGR and PD.
5. Keratinized tissue height (KTH): Calculated from distance amid the most apical point of GM to mucogingival junction (MGJ).
6. Gingival thickness (GT): GT is assessed in attached gingiva 3 mm apical to the GM using #15 endodontic reamer with rubber stop. After anesthetizing, the gingival surface 2–3 mm below the margin of gingiva is pierced at 90° angle with slight pressure until hard tissue is reached. Rubber stop on reamer is slid until close contact with gingiva. Once the reamer is taken away, the distance between the tip of the reamer and the inner border of the rubber stop will be measured and rounded to the closest mm with a graduated scale.
7. Percentage of root coverage: 6 months after treatment.

Postoperative recession depth − preoperative recession depthPreoperative recession depth×100.

### 3.3. Treatment Protocol

Prior to commencing this study, the aims and structure were conveyed to the patient and provided with informed consent. Details regarding the main complaint, the current illness history, and relevant dental, medical, family, medication, personal background, the condition of the gums, as well as periodontal health, were recorded in this case proforma. Patients were assessed under sufficient lighting using a mouth mirror, William’s graduated periodontal probe, and cotton gauze.

In the initial therapy, each patient began with treatment that includes guidance on oral hygiene, scaling, root planing, and a professional cleaning using a rubber cup and gentle polishing paste. Coronoplasty was carried out on buccal prominences and in cases where occlusal trauma was identified. Modified Stillman’s brushing technique was recommended for areas with recession‐type defects for reducing trauma while brushing. Instructions on control of plaque were reiterated until patients reached a full mouth plaque score of less than 20% and full mouth modified SBI of below 20%. A complete hemogram was taken prior to the procedure. Customized acrylic stents were prepared to measure PD from the CEJ at baseline and after 6 months [9].

Before the surgical procedure, patients were asked to rinse their mouths with a 0.2% chlorhexidine gluconate solution for 1 min. The surgical protocol highlights the importance of complete asepsis and controlling infection. Following injecting the local anesthesia (2% lidocaine, epinephrine 1:100000), the exposed root surface and potential depth were smoothed using curettes in removing the soft tooth structure, undercuts, ridges, plaque, and calculus. Following the instrumentation process, the root surfaces were flushed with a saline solution to help eliminate any loose fragments from the surgical area and defect.

### 3.4. Surgical Technique: (Figures 1–15)

Following the cleaning of the surgical site, draping of the patient, and application of local anesthesia, root planing and smoothing were carried out using Gracey curettes (2R2L and 4R4L) and a polishing bur. Restoration of NCCL was achieved employing RMGIC. The area to be restored was isolated, and then, RMGIC was mixed following the manufacturer’s guidelines, using equal parts powder and liquid (1:1). It was placed into the cavity using a plastic filling instrument and pressed against the cavity walls. The material is cured with light for about 40 s. Next, the restoration is shaped and smoothed under water spray with tungsten carbide burs, followed by polishing (Figures 1 and 10).

**Figure 1:**
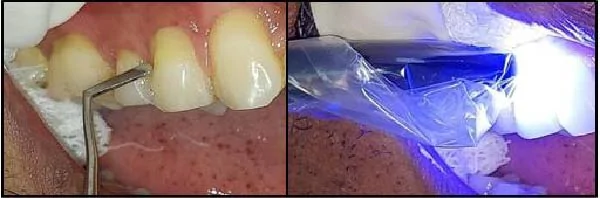
Restoration of RMGIC on the NCCL followed by light curing‐test group.

**Figure 2:**
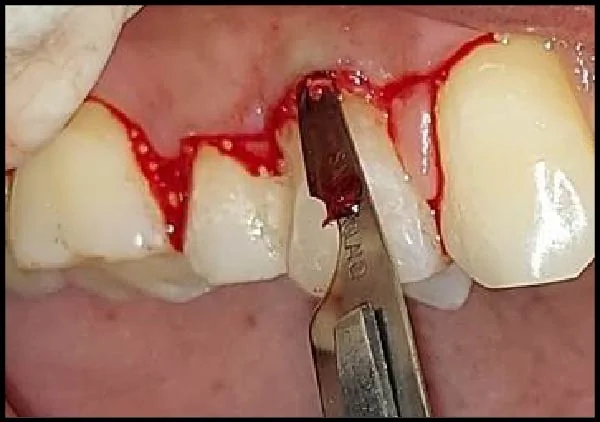
Intrasulcular and oblique incision given‐test group.

**Figure 3:**
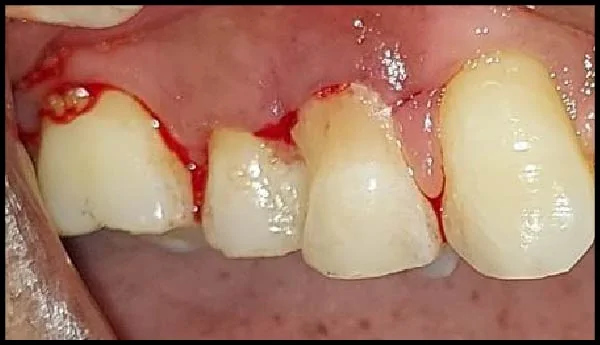
Flap reflection (split‐full‐split)‐test group.

**Figure 4:**
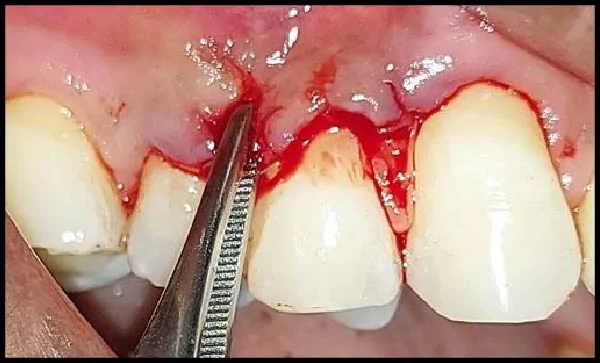
Coronally advancing the flap without tension‐test group.

**Figure 5:**
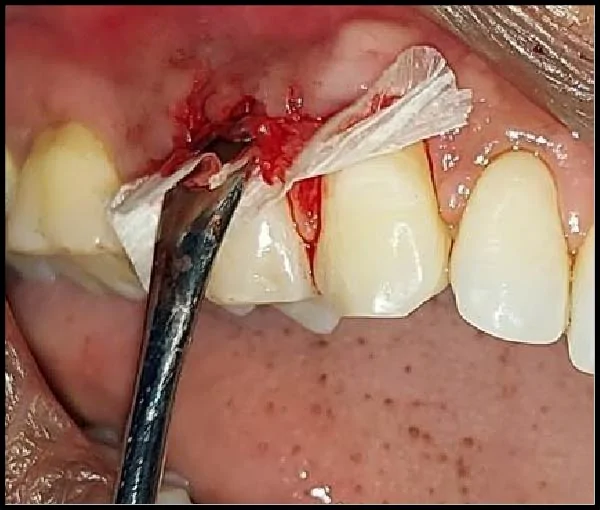
Placement of amnion membrane‐test group.

**Figure 6:**
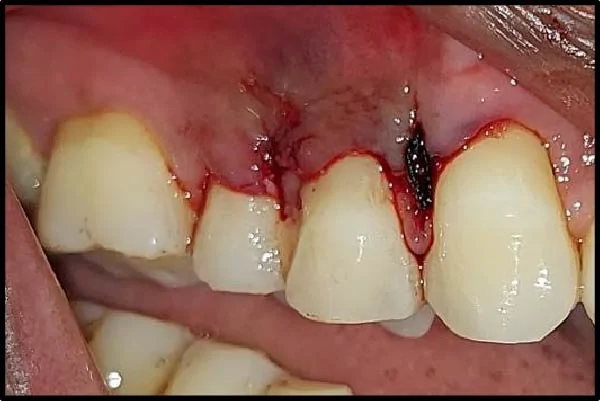
4–0 Sutures placed and stabilized‐test group.

**Figure 7:**
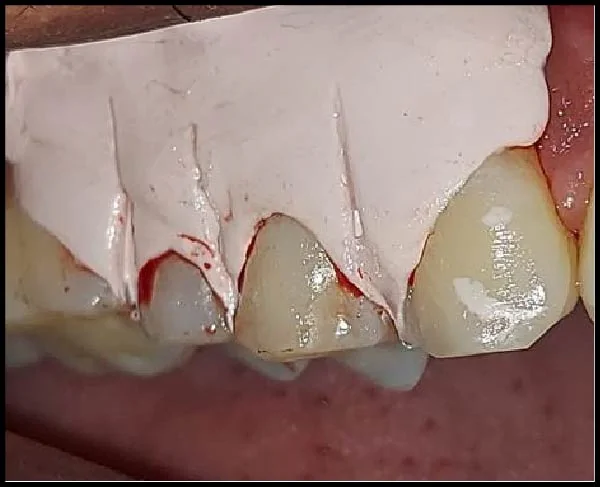
Periodontal pack placed‐test group.

**Figure 8:**
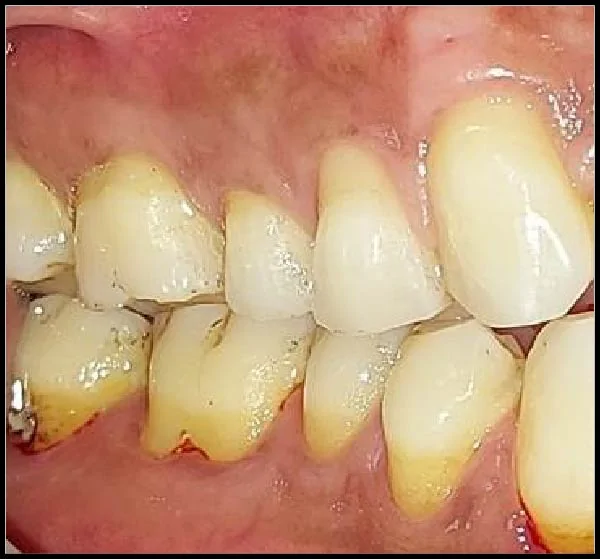
Preoperative‐test group.

**Figure 9:**
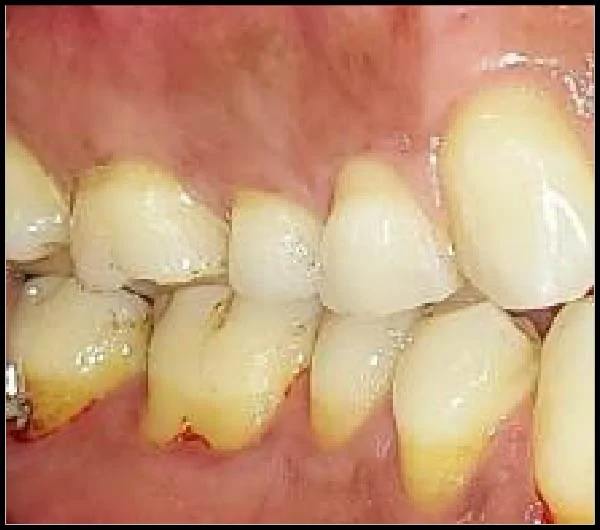
Months postoperative‐test group.

**Figure 10:**
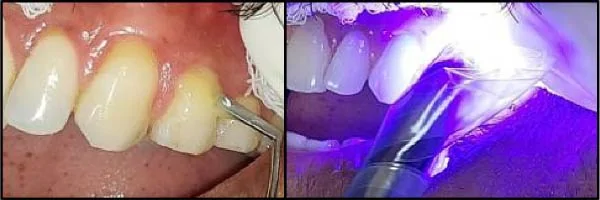
Restoration of RMGIC on the NCCL followed by light curing‐control group.

**Figure 11:**
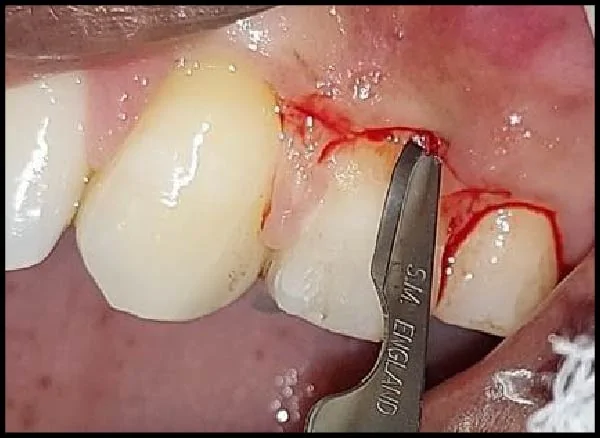
Intrasulcular and oblique incision given‐control group.

**Figure 12:**
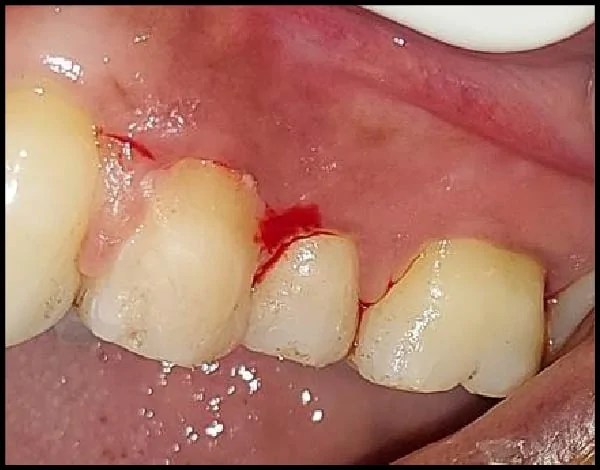
Flap reflection (split‐full‐split)‐control group.

**Figure 13:**
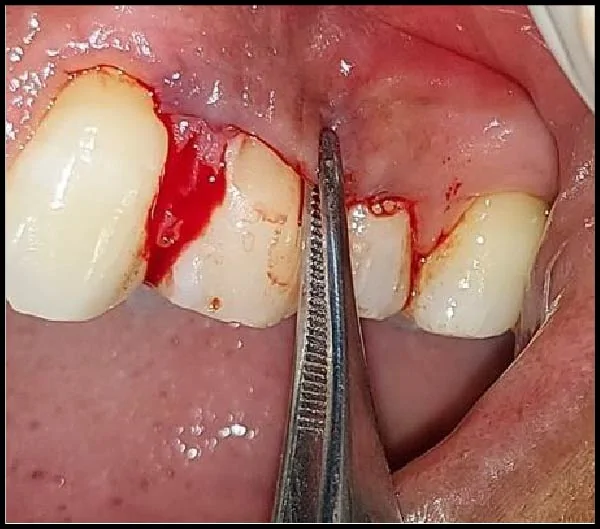
Coronally advancing the flap without tension‐control group.

**Figure 14:**
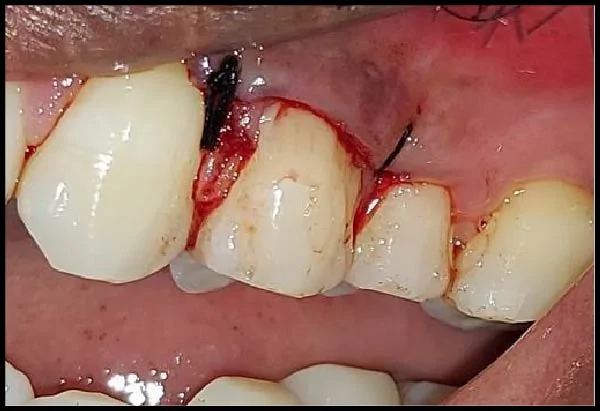
4–0 sutures placed and stabilized‐control group.

**Figure 15:**
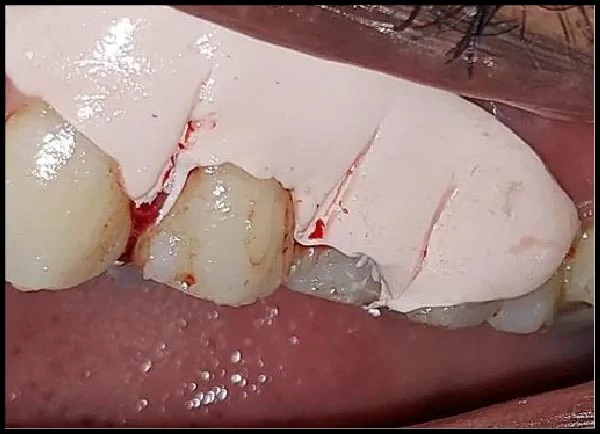
Periodontal pack placed‐control group.

The whole length of NCCL was repaired to ensure that the tooth’s original shape is restored.

A No. 15 blade envelope MCAF, as proposed by Zucchelli et al. [9] and De Sanctis et al. [10], was used and included the following incisions. Horizontal incisions were made at the level of CEJ, along with sulcular incisions on the buccal side of involved teeth (Figures 2 and 11). A partial‐full‐partial thickness flap was performed (Figures 3 and 12). The interdental area had the papilla deepithelialized. Flap was reflected in full thickness apical to marginal gingiva to enable the periodontium to cover the avascular surface of the root. To facilitate coronal repositioning of the flap, it was elevated partially past the MGJ (Figures 4 and 13). Root and NCCL lesion was planed using a finishing bur and curettes until the root surface is clean and smooth.

In the test group, the amnion membrane was trimmed by an additional 2 mm on each side to match the dimensions of the treatment area. The membrane was applied directly onto the root surface (Figure 5). Due to the adhesive nature of the amnion, the membrane did not need to be sutured. The flap was positioned coronally and secured using a sling suture without any tension with a 4–0 mersilk suture [11] (Figures 6 and 14). The final placement of the flap margin was set 3–4 mm above the CEJ of the tooth. Right after the surgery, a periodontal dressing was put on to surgical area (Figures 7 and 15).

### 3.5. Postoperative Care: (Figures 8, 9, 16, and 17)

External ice pack application was utilized for 30 min at a time, alternating on and off, and patients were advised to maintain their regular oral hygiene at home for the following 24 h. Antibiotics were prescribed. A nonsteroidal anti‐inflammatory tablet was prescribed on a case‐by‐case basis as needed. Patients were informed to persist with their regular home hygiene practices, except for the area that was operated on, where tooth brushing should be avoided for the initial 2–3 weeks postsurgery, and plaque control was managed with a 0.2% chlorhexidine gluconate mouthwash, eventually transitioning to a soft toothbrush while employing Charter’s brushing technique. The patients were scheduled for a follow‐up appointment 2 weeks later to have the stitches removed. Follow‐up visits were arranged 6 months after surgery, during which oral prophylaxis was conducted. Clinical measurements taken prior to the operation were reassessed at baseline and 6 months succeeding the treatment (Figures 8, 9, 16, and 17).

**Figure 16:**
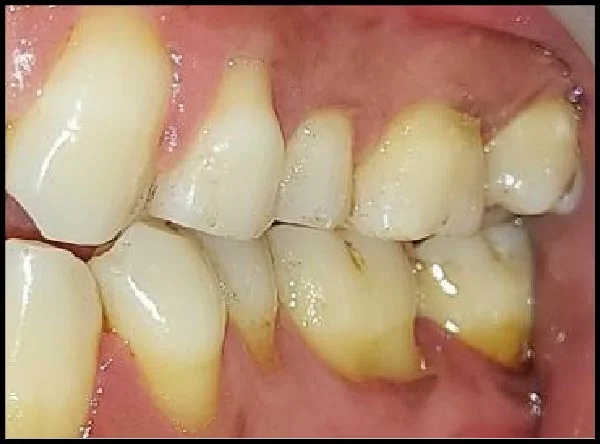
Preoperative‐control group.

**Figure 17:**
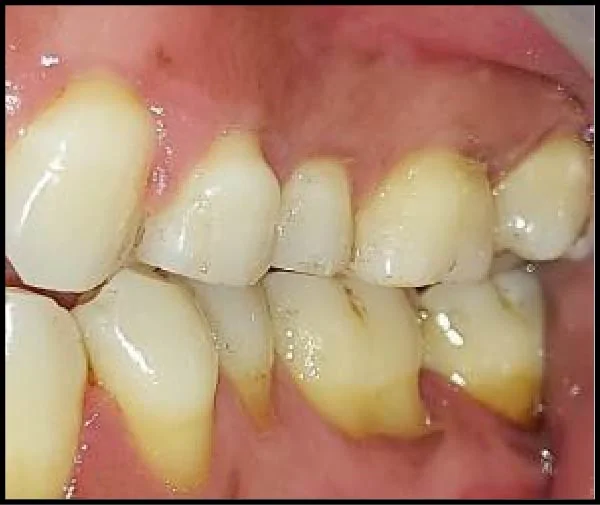
6 months postoperative‐control group.

### 3.6. Statistical Analysis

Data were assessed for normality before the analysis. The paired *t*‐test was used for intragroup comparisons of continuous variables, whereas the independent *t*‐test was used for intergroup comparisons between the two treatment modalities. A *p*‐value <0.05 was considered statistically significant.

## 4. Results

A total of 15 systemically healthy participants were included in the research. All participants finished the study without any dropouts, and there were no reported adverse events during the follow‐up periods. In this research, the test groups received treatment with the amnion membrane combined with MCAF and RMGIC, while the control group was treated solely with MCAF and RMGIC.

The CONSORT flow chart is included in Figure 18.

**Figure 18:**
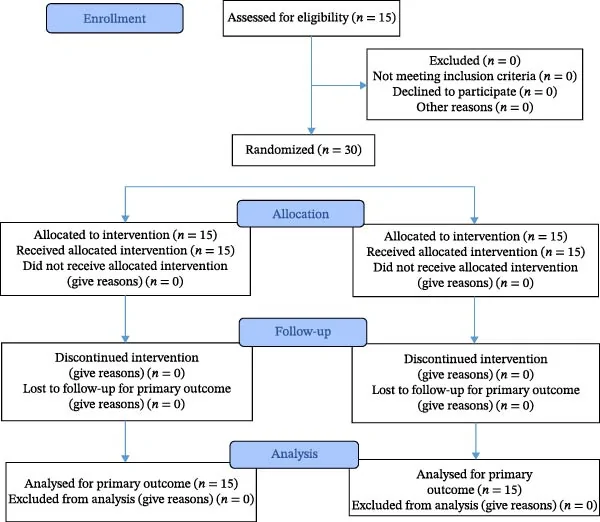
Consort flow chart.

### 4.1. PI

At baseline, both test and control groups exhibited significantly higher mean values compared to their respective 6‐month follow‐up assessments (test:difference = 1.43, *p* < 0.001; control: difference = 1.07, *p* < 0.001). Between‐group comparison at baseline showed no statistically significant difference (mean difference = 0.07; *p* = 0.605). However, at 6 months, test group demonstrated significantly higher mean value than the control group (mean difference = 0.43; *p* = 0.003). The overall change favored the test group (mean difference = 0.36; *p* < 0.001) (Table 1).

### 4.2. SBI

Test group showed significant reduction from baseline to 6 months (mean difference = 2.04; *p* < 0.001), as did control group (mean difference = 1.27; *p* < 0.001). Between‐group comparison at baseline was nonsignificant (0.25; *p* = 0.384), while at 6 months, the test group had a significantly greater mean (0.53; *p* < 0.001). The overall difference in mean change favored the test group (21.59; *p* < 0.001), (Table 2) indicating a statistically significant treatment effect.

**Table 2:** Comparison of mean sulcular bleeding index scores between the two groups at baseline and after 6 months.

Group	Time point	*N*	Mean ± SD	Mean difference ± SD	*t*	*p*‐Value
Intragroup comparison:						
Test	At baseline	15	2.65 ± 0.11	2.04 ± 0.11	74.85	<0.001 ^∗^
	After 6 months	15	0.61 ± 0.09			
Control	At baseline	15	2.4 ± 0.17	1.27 ± 0.06	79.48	<0.001 ^∗^
	After 6 months	15	1.13 ± 0.22			
Intergroup comparison:						
Test	At baseline	15	2.65 ± 0.11	−0.25 ± 0.14	−7.05	0.345
Control	At baseline	15	2.4 ± 0.17			
Test	After 6 months	15	0.61 ± 0.09	0.53 ± 0.19	10.49	<0.001 ^∗^
Control	After 6 months	15	1.13 ± 0.22			
Test	Mean difference	15	2.04 ± 0.11	−0.77 ± 0.14	−21.59	<0.001 ^∗^
Control	Mean difference	15	1.27 ± 0.06			

*Note*:  ^∗^statistically significant difference (*p*  < 0.05).

### 4.3. PD

The test group exhibited a significant reduction from baseline to 6 months (mean difference = 1.67; *p* < 0.001), as did control group (mean difference = 0.93; *p* < 0.001). Between‐group comparison at baseline showed a nonsignificant difference (0.20; *p* = 0.189). At 6 months, test group had a significantly greater mean than control (0.53; *p* = 0.001). The overall mean difference in change favored the test group (0.73), which is statistically significant (*p* < 0.001) (Table 3).

**Table 3:** Comparison of mean probing depth (PD) scores between the two groups at baseline and after 6 months.

Group	Time point	*N*	Mean ± SD	Mean difference ± SD	*t*	*p*‐Value
Intragroup comparison						
Test	At baseline	15	2.67 ± 0.49	1.67 ± 0.49	13.23	<0.001 ^∗^
	After 6 months	15	1 ± 0			
Control	At baseline	15	2.47 ± 0.52	0.93 ± 0.46	7.90	<0.001 ^∗^
	After 6 months	15	1.53 ± 0.52			
Intergroup comparison						
Test	At baseline	15	2.67 ± 0.49	−0.2 ± 0.56	−1.38	0.189
Control	At baseline	15	2.47 ± 0.52			
Test	After 6 months	15	1 ± 0	0.53 ± 0.52	4.00	0.001 ^∗^
Control	After 6 months	15	1.53 ± 0.52			
Test	Mean difference	15	1.67 ± 0.49	−0.73 ± 0.46	−6.21	<0.001 ^∗^
Control	Mean difference	15	0.93 ± 0.46			

*Note*:  ^∗^statistically significant difference (*p*  < 0.05).

### 4.4. RCAL

Test group presented significant decrease from baseline to 6 months (mean difference = 3.87; *p* < 0.001), as did control group (mean difference = 2.6; *p* < 0.001). At baseline, no significant difference were observed between groups (0.00; *p* = 1). At 6 months, test group had a significantly greater mean than control (1.27; *p* < 0.001). The overall mean change also favored the test group (mean difference = 1.27; *p* < 0.001) (Table 4).

**Table 4:** Comparison of mean relative clinical attachment level (RCAL) scores between the two groups at baseline and after 6 months.

Group	Time point	*N*	Mean ± SD	Mean difference ± SD	*t*	*p*‐Value
Intragroup comparison						
Test	At baseline	15	8.27 ± 0.8	3.87 ± 0.74	20.15	<0.001 ^∗^
	After 6 months	15	4.4 ± 0.51			
Control	At baseline	15	8.27 ± 0.88	2.6 ± 0.63	15.92	<0.001 ^∗^
	After 6 months	15	5.67 ± 0.82			
Intergroup comparison						
Test	At baseline	15	8.27 ± 0.8	0 ± 1.07	0.00	1
Control	At baseline	15	8.27 ± 0.88			
Test	After 6 months	15	4.4 ± 0.51	1.27 ± 0.96	5.10	<0.001 ^∗^
Control	After 6 months	15	5.67 ± 0.82			
Test	Mean difference	15	3.87 ± 0.74	−1.27 ± 0.96	−5.10	<0.001 ^∗^
Control	Mean difference	15	2.6 ± 0.63			

*Note*:  ^∗^statistically significant difference (*p*  < 0.05).

### 4.5. Relative Gingival Recession

At 6 months, both groups showed significant reductions from baseline (test: 2.2, *p* < 0.001; control: 1.67, *p* < 0.001). Between‐group comparison revealed no significant baseline difference (0.2, *p* = 0.384), but the test group showed significantly greater improvement postoperatively (0.733, *p* = 0.001; overall mean difference: 0.53, *p* = 0.027) (Table 5).

**Table 5:** Comparison of mean relative gingival recession (RGR) scores between the two groups at baseline and after 6 months.

Group	Time Point	*N*	Mean ± SD	Mean difference ± SD	*t*	*p*‐Value
Intragroup comparison:						
Test	At baseline	15	5.6 ± 0.63	2.2 ± 0.41	20.58	<0.001** ^∗^**
	After 6 months	15	3.4 ± 0.51			
Control	At baseline	15	5.8 ± 0.77	1.67 ± 0.62	10.46	<0.001** ^∗^**
	After 6 months	15	4.13 ± 0.52			
Intergroup comparison:						
Test	At baseline	15	5.6 ± 0.63	0.2 ± 0.86	0.90	0.384
Control	At baseline	15	5.8 ± 0.77			
Test	After 6 months	15	3.4 ± 0.51	0.73 ± 0.7	4.04	0.001 ^∗^
Control	After 6 months	15	4.13 ± 0.52			
Test	Mean difference	15	2.2 ± 0.41	−0.53 ± 0.83	−2.48	0.027 ^∗^
Control	Mean difference	15	1.67 ± 0.62			

*Note*:  ^∗^statistically significant difference (*p*  < 0.05).

### 4.6. RW

Test group showed significant decrease from baseline to 6 months (mean difference = 2.6; *p* < 0.001), as did the control group (mean difference = 0.4; *p* = 0.009). At baseline, the between‐group difference was nonsignificant (−0.47; *p* = 0.068). After 6 months, the test group had significantly greater mean (1.73; *p* = 0.001). The overall change favored test group, which exhibited significantly higher mean difference (−2.2; *p* < 0.001) (Table 6).

**Table 6:** Comparison of mean recession width (RW) scores between the two groups at baseline and after 6 months.

Group	Time point	*N*	Mean ± SD	Mean difference ± SD	*t*	*p*‐Value
Intragroup comparison:						
Test	At baseline	15	3.8 ± 0.68	2.6 ± 1.5	—	<0.001** ^∗^**
	After 6 months	15	1.2 ± 1.52			
Control	At baseline	15	3.33 ± 0.49	0.4 ± 0.51	—	0.009
	After 6 months	15	2.93 ± 0.26			
Intergroup comparison:						
Test	At baseline	15	3.8 ± 0.68	−0.47 ± 0.92	−1.97	0.068
Control	At baseline	15	3.33 ± 0.49			
Test	After 6 months	15	1.2 ± 1.52	1.73 ± 1.62	4.13	0.001 ^∗^
Control	After 6 months	15	2.93 ± 0.26			
Test	Mean difference	15	2.6 ± 1.5	−2.2 ± 1.7	−5.02	<0.001 ^∗^
Control	Mean difference	15	0.4 ± 0.51			

*Note*:  ^∗^statistically significant difference (*p*  < 0.05).

### 4.7. KTH

From baseline to 6 months, the test group showed a significant increase in mean values (difference = 2.07; *p* < 0.001), as did the control group (difference = 1; *p* < 0.001). Baseline comparison revealed no significant difference between groups (0.4; *p* = 0.233). After 6 months, the test group had significantly higher mean values (1.47; *p* < 0.001). The overall mean difference in change favored test group (1.07; *p* < 0.001) (Table 7).

**Table 7:** Comparison of mean keratinized tissue height (KTH) scores between the two groups at baseline and after 6 months.

Group	Time point	*N*	Mean ± SD	Mean difference ± SD	*t*	*p*‐Value
Intragroup comparison						
Test	At baseline	15	3.53 ± 0.99	−2.07 ± 0.59	−13.48	<0.001 ^∗^
	After 6 months	15	5.6 ± 0.83			
Control	At baseline	15	3.13 ± 0.74	−1 ± 0.55	−5.6	<0.001 ^∗^
	After 6 months	15	4.13 ± 0.74			
Intergroup comparison						
Test	At baseline	15	3.53 ± 0.99	−0.4 ± 1.24	−1.25	0.233
Control	At baseline	15	3.13 ± 0.74			
Test	After 6 months	15	5.6 ± 0.83	−1.47 ± 1.06	−5.36	<0.001 ^∗^
Control	After 6 months	15	4.13 ± 0.74			
Test	Mean difference	15	−2.07 ± 0.59	1.07 ± 0.59	6.96	<0.001 ^∗^
Control	Mean difference	15	−1 ± 0			

*Note*:  ^∗^statistically significant difference (*p*  < 0.05).

### 4.8. Gingival Thickness (GT)

The test group showed a significant increase in mean values from baseline to 6 months (difference = 1.13; *p* < 0.001), while the control group values remained comparable. Baseline comparison revealed no significant difference among groups. However, at 6 months, the test group had a significantly higher mean (difference = 1.13; *p* < 0.001). The overall mean change favored test group, which demonstrated a significantly greater improvement (mean difference = 1.13; *p* < 0.001) (Table 8).

**Table 8:** Comparison of mean gingival thickness (GT) scores between the two groups at baseline and after 6 months.

Group	Time point	*N*	Mean ± SD	Mean difference ± SD	*t*	*p*‐Value
Intragroup comparison						
Test	At baseline	15	1.33 ± 0.49	−1.13 ± 0.35	−12.48	<0.001** ^∗^**
	After 6 months	15	2.47 ± 0.64	—	—	—
Control	At baseline	15	1.33 ± 0.49	—	—	**—**
	After 6 months	15	1.33 ± 0.49	—	—	—
Intergroup comparison						
Test	At baseline	15	1.33 ± 0.49	—	—	**—**
Control	At baseline	15	1.33 ± 0.49	—	—	—
Test	After 6 months	15	2.47 ± 0.64	−1.13 ± 0.35	−12.48	<0.001 ^∗^
Control	After 6 months	15	1.33 ± 0.49	—	—	—
Test	Mean difference	15	−1.13 ± 0.35	1.13 ± 0.35	12.48	<0.001** ^∗^**
Control	Mean difference	15	0 ± 0	—	—	—

*Note*:  ^∗^statistically significant difference (*p*  < 0.05).

### 4.9. Mean Root Coverage (MRC) and Root Coverage Esthetic Score (RES)

Test group showed significantly higher MRC (difference = 11.08%; *p* = 0.003) and higher GM levels (difference = 2.2; *p* = 0.006). Differences in marginal tissue contour (0.2; *p* =  0.082), MGJ alignment (0.2; *p* = 0.082), and gingival color were not statistically significant. Soft tissue texture was comparable between the groups. Total RES score was significantly greater in test group (difference = 2.6; *p* = 0.009), (Table 9) indicating superior overall clinical outcomes.

**Table 9:** Comparison of mean percentage root coverage and mean root coverage esthetic score (RES) between the two groups.

Variable	Group	*N*	Mean ± SD	Mean difference ± SD	*t*	*p*‐Value
Mean root coverage (%)	Test	15	39.3 ± 5.5	11.08 ± 11.83	3.63	0.003 ^∗^
	Control	15	28.22 ± 8.26			
Level of gingival margin	Test	15	5 ± 1.46	−2.2 ± 2.65	−3.21	0.006 ^∗^
	Control	15	2.8 ± 1.78			
Marginal tissue contour	Test	15	1 ± 0	−0.2 ± 0.41	−1.87	0.082
	Control	15	0.8 ± 0.41			
Soft tissue texture	Test	15	1 ± 0	—	—	—
	Control	15	1 ± 0			
Mucogingival junction alignment	Test	15	1 ± 0	−0.2 ± 0.41	−1.87	0.082
	Control	15	0.8 ± 0.41			
Gingival color	Test	15	1 ± 0	—	—	—
	Control	15	1 ± 0			
Total	Test	15	9 ± 1.46	−2.6 ± 3.31	−3.04	0.009 ^∗^
	Control	15	6.4 ± 2.5			

*Note*:  ^∗^statistically significant difference (*p*  < 0.05).

## 5. Discussion

Gingival recession, characterized by apical displacement of the GM, leads to root exposure, hypersensitivity, and esthetic concerns. Miller’s (1985) classification remains widely used in root coverage studies, emphasizing recession extent, interdental tissue loss, and bone involvement [12]. Although CAF with CTG has long been the gold standard for root coverage, it often involves donor‐site morbidity and surgical complexity [8]. Recently, the AM, rich in growth factors and ECM proteins, has shown promise in enhancing periodontal regeneration. This study evaluated the efficacy of MCAF combined with RMGIC and AM using MRC percentage and RES scores. Results support AM as a viable alternative to traditional grafts, offering comparable outcomes with fewer complications [13]. Additionally, NCCLs—noncarious enamel/dentin defects near the CEJ—are implicated in nearly 50% of recession cases, highlighting their role in clinical management [14].

### 5.1. PI Scores

Both the test (MCAF + RMGIC + AM) and control (MCAF + RMGIC) groups exhibited significant reductions in PI scores over 6 months.Control group showed mean reduction of 1.07 (*p* < 0.001), while test group demonstrated greater decrease of 1.43 (*p* < 0.001), indicating enhanced plaque control with AM. Baseline PI scores were comparable (*p* = 0.605), but at 6 months, the test group had a significantly lower score, with a between‐group difference of 0.43 (*p* = 0.003). The overall intergroup PI reduction favored the test group (0.36; *p* < 0.001), suggesting the adjunctive benefit of AM. These outcomes align with previous studies, including systematic reviews and meta‐analyses, which highlight AM’s regenerative potential and long‐term plaque control. Despite potential plaque retention due to altered gingival contours, studies such as those of Rehan et al. [11], affirm similar PI improvements across biomaterials, reinforcing the need for rigorous oral hygiene. AM appears to enhance periodontal outcomes with manageable clinical considerations [15].

### 5.2. Bleeding Scores

Although baseline SBI scores were similar between groups (0.25; *p* = 0.384), test group demonstrated significantly higher reduction after 6 months (difference = 0.53; *p* < 0.001). The mean SBI change favored the test group (21.59; *p* < 0.001), suggesting a unique influence of AM on bleeding control. These findings align with prior studies by Dias et al. [16] showing SBI’s sensitivity in disease progression and supporting AM’s adjunctive benefits in enhancing periodontal health.

### 5.3. PD

Significant reductions were seen over 6 months in both the control (MCAF + RMGIC) and test (MCAF + RMGIC + AM) groups. Test group showed higher mean PD reduction (1.67; *p* < 0.001) compared to control group (0.93; *p* < 0.001), indicating enhanced periodontal healing with AM. Baseline PD values were comparable (*p* = 0.189), but at 6 months, the test group presented significantly lower mean PD (*p* = 0.001), with intergroup difference of 0.73 (*p* < 0.001). These results suggest a measurable benefit from AM in improving clinical outcomes. Findings are supported by previous studies, including Saleh et al. [17], who reported improved PD reduction with adjunctive regenerative materials. Similar trends were noted by Van Dyke et al. [18] and Rehan et al. [11], further validating AM’s role in enhancing periodontal repair via anti‐inflammatory and epithelialization‐promoting properties. Systematic reviews confirm AM’s efficacy in root coverage procedures and support its integration into recession therapy [19]. Additionally, evidence suggests a favorable tissue response to glass ionomer restorations in NCCL management [3], indirectly contributing to PD stability. Collectively, these findings position AM as a promising biomaterial for optimizing clinical parameters in mucogingival therapy.

### 5.4. RCAL

Significant improvements were seen in both the control (MCAF + RMGIC) and test (MCAF + RMGIC + AM) groups over 6 months. The test group showed a greater gain (3.87 mm) than the control (2.6 mm), both statistically significant (*p* < 0.001). At baseline, RCAL values were similar (*p* = 1); however, at 6 months, the test group exhibited a significantly higher improvement (1.27 mm; *p* < 0.001). These findings suggest that AM may enhance periodontal attachment outcomes. They align with studies highlighting the regenerative properties of AM, including growth factor release and anti‐inflammatory effects. Reviews by Karmakar and Prakash [20] and Liu et al. [21] support its utility in improving CAL, comparable to or exceeding results achieved with CTG. Randomized trials by Payne et al. [22] and clinical data from Santamaria et al. [4] further validate the role of adjunctive materials like AM in achieving stable attachment gains, especially in NCCL‐associated recession cases.

### 5.5. RGR

Both groups demonstrated significant reductions in scores over 6 months (*p* < 0.001). While baseline values were comparable, a significant intergroup difference were seen at 6 months (*p* = 0.001), with test group (MCAF + RMGIC + AM) achieving a greater mean reduction (2.2 mm) than the control (1.67 mm), yielding a statistically significant difference of 0.53 mm (*p* = 0.027). These findings align with studies indicating enhanced root coverage when connective tissue grafts, collagen matrix, or PRF are combined with CAF [23]. Evidence from Nath et al. [24] and Rehan et al. [11] supports the superior efficacy of AM in reducing recession dimensions, attributed to its bioactivity and regenerative properties. Improvements in GM alignment and surface texture per RES criteria further validate the observed benefits. This reinforces the clinical potential of AM as a reliable adjunct in mucogingival procedures for managing recession defects.

### 5.6. RW

Significant reductions were seen for both test (MCAF +  RMGIC + AM) and control (MCAF + RMGIC) groups. Test group achieved higher reduction (2.6 mm; *p* < 0.001) in comparison to control (0.4 mm; *p* = 0.009), with a statistically significant intergroup difference of 2.2 mm (*p* < 0.001), indicating the added benefit of AM. These findings are consistent with meta‐analyses by Lubaib et al. [15] showing that AM, when combined with CAF, yields comparable or superior RW reduction to CTG. The control group’s limited improvement highlights RMGIC’s limited role beyond hypersensitivity management. Further, in a meta‐analysis by Abdel‐Fatah and Saleh [19], CAF + AM has been shown to outperform PRF and collagen membranes, delivering enhanced esthetics and more effective root coverage. However, AM’s limited impact on keratinized gingival width (WKG) underscores the importance of combining regenerative strategies when tissue augmentation is the goal. These outcomes reinforce AM’s clinical utility in improving the outcomes of flap‐based root coverage procedures.

### 5.7. Mean KTH

The revealed significant increase in KTH in both test (MCAF + RMGIC + AM) and control (MCAF + RMGIC) groups over 6 months. Test group showed greater gain (2.07 mm; *p* < 0.001) than control group (1.00 mm; *p* < 0.001), with statistically significant intergroup difference of 1.47 mm (*p* < 0.001). Although baseline KTH values were similar (*p* = 0.233), the test group demonstrated a mean difference of 1.07 mm in favor of AM (*p* < 0.001), highlighting its regenerative potential. These findings align with studies by Adibrad et al. [25] and Nalbantoglu and Yanık [26], which emphasize the importance of ≥2 mm of keratinized gingiva for optimal periodontal health and long‐term stability. Results from Bolla et al. [27], Carcuac et al. [28], and Terenzi et al. [29] further support the role of AM in increasing KTH comparably to FGG, yet with reduced invasiveness. AM’s ability to promote keratinized tissue formation without donor site morbidity positions it as an effective, patient‐friendly alternative to traditional grafting procedures.

### 5.8. GT

Significant improvement was seen in the test group (MCAF +  RMGIC + AM) compared to control (MCAF + RMGIC) over 6 months. While control group showed no appreciable change, test group exhibited statistically significant increase of 1.13 mm (*p* < 0.001). At baseline, both groups had comparable GT values (*p* = 1.0), but the test group displayed a significantly greater GT after 6 months, with an intergroup difference of 1.13 mm (*p* < 0.001). These findings support AM’s role in augmenting gingival tissue, likely due to its bioactive properties that enhance fibroblast proliferation, angiogenesis, and reduce inflammation. The results align with previous findings by Kumar et al. [30], who observed lasting improvements in GT following AM use with CAF. A meta‐analysis by Lubaib et al. [15] further reinforced AM’s efficacy compared to CAF alone or with CTG. Although SCTG remains the gold standard, AM offers comparable outcomes with reduced surgical morbidity. Additional studies by Singh et al. [31] and Santamaria et al. [32] also demonstrate GT enhancement when AM is used adjunctively. Collectively, these findings highlight AM’s regenerative potential and its relevance as a minimally invasive alternative to periodontal soft tissue augmentation.

### 5.9. Mean Percentage Root Coverage

Mean percentage root coverage is significantly greater in test group (MCAF + RMGIC + AM) compared to the control (MCAF + RMGIC), with an 11.08% difference (*p* = 0.003). This supports AM’s regenerative potential, attributed to its proepithelialization effects, anti‐inflammatory properties, and presence of growth factors such as VEGF and PDGF. The findings are consistent with prior studies, including Sharma and Yadav [33], who reported 70.2% root coverage with AM, comparable to CTG. Unlike CTG, AM avoids donor‐site morbidity, offering a less invasive alternative. However, Singh et al. [31] reported no significant difference when AM was used with PRF, suggesting variability depending on adjunctive materials. A trial by Santamaria et al. [32] further emphasized that root coverage outcomes improve in NCCL‐associated sites when restorations are present. These results reinforce AM’s role in improving root coverage outcomes, particularly in challenging clinical scenarios.

### 5.10. Root Coverage Esthetic Score (RES)

The test group demonstrated superior esthetic outcomes, reflected in significantly higher overall RES scores (difference = 2.6; *p* = 0.009), primarily driven by improvements in GM levels (*p* = 0.006). While enhancements in marginal tissue contour and MGJ alignment were noted, they were not statistically significant (*p* = 0.082). These findings align with recent literature emphasizing AM’s regenerative properties, including anti‐scarring effects and natural tissue integration. Studies by Law et al. [34] and others have shown that AM sustains esthetic results over time and offers advantages over CTG and PRF, such as reduced surgical complexity and donor‐site morbidity. While CTG remains the gold standard for root coverage, AM demonstrates comparable improvements in the esthetic parameters and keratinized tissue thickness. Nonetheless, as the present study is limited by a small sample size and short follow‐up, further long‐term, multicentric trials are recommended to confirm the durability and consistency of these esthetic benefits.

## 6. Limitations

• The relatively small sample size and single‐center design may limit the generalizability of the results.
• In addition, the 6‐month follow‐up period does not allow the assessment of long‐term treatment stability.
• Future multicenter studies with larger sample sizes and longer follow‐up periods are recommended to validate and extend the present findings.

## 7. Conclusion

This study evaluated the clinical efficacy of combining MCAF with RMGIC and AM in managing gingival recession associated with NCCL. This randomized, split‐mouth design revealed that the test group achieved significantly superior outcomes compared to the control group The inclusion of AM offered regenerative advantages due to its anti‐inflammatory, proepithelialization, and growth factor‐rich matrix, facilitating improved wound healing without donor‐site morbidity. RMGIC contributed functional benefits through chemical adhesion, fluoride release, and structural support for restoring NCCLs, enhancing both biological and mechanical stability. While short‐term outcomes are encouraging, long‐term, multicenter trials are recommended to validate these results and refine protocols for broader clinical adoption. This approach thus offers a compelling option for managing recession defects with functional and esthetic predictability.

## Author Contributions

Conceptualization, supervision, writing – review and editing: S. Sanidhya, Deepa G.Kamath, and Mranali K. Shetty. Data curation, format analysis: S. Sanidhya and Deepa G. Kamath. Investigation, writing – original draft: S. Sanidhya. Methodology: S. Sanidhya, Deepa G. Kamath, Mranali K. Shetty, and N. Srikant. Project administration, resources: S. Sanidhya, Deepa G. Kamath, and Aradhya Sinha. Software: S. Sanidhya and N. Srikant. Validation, visualization: Deepa G. Kamath and Mranali K. Shetty.

## Funding

No funding was received for this manuscript.

## Consent

Written informed consent for the publication of this manuscript and accompanying images was obtained from the participants.

## Conflicts of Interest

The authors declare no conflicts of interest.

## Data Availability

The datasets generated and/or analyzed during the current study are available from the corresponding author upon reasonable request.
